# Marital coitus in Bangladesh in the 2010s: trends and sociodemographic determinants

**DOI:** 10.7189/jogh.15.04139

**Published:** 2025-06-27

**Authors:** Md Tazvir Amin, Md Mahabubur Rahman, Shusmita Hossain Khan, Afsana Bhuiyan, Mizanur Rahman, Nurul Alam, M Moinuddin Haider

**Affiliations:** 1 Health Systems and Population Studies Division, icddr,b, Dhaka, Bangladesh; 2 Data for Impact, University of North Carolina at Chapel Hill, North Carolina, USA; 3 London North West University Healthcare NHS Trust, London, UK

## Abstract

**Background:**

In Bangladesh, marriage is the sole socially accepted context for sexual intimacy or coitus between partners. Over the past decade, the predictors of coitus in the country have undergone notable changes, including progress in socioeconomic conditions, health behaviours, nutritional status, and contraceptive practices. Despite these changes, there exists no empirical evidence regarding patterns of coitus in Bangladesh, leaving a critical gap in our understanding of sexual behaviour in the country. We aimed to examine the changes and associated predictors of marital coitus among married couples in Bangladesh from 2011 to 2017–18 at the national level.

**Methods:**

We used data from three nationally representative Bangladesh Demographic and Health Surveys (BDHSs) conducted in 2011, 2014, and 2017–18, whereby we included currently married women of reproductive age (CMWRA) living with their husbands who were not infecund or had not reached menopause in our analysis (n = 38 853). By pooling their data, we examined the changes in weekly marital coitus (WMC), queried within the survey through the question ‘Was a CMWRA involved in sexual activity in the last four weeks preceding the survey?’. The determinant analysis included data from the 2017–18 BDHS (n = 13 686). We used bivariate analysis and mixed-effect logistic regression to investigate the changes and predictors of WMC among CMWRAs.

**Results:**

Compared to 2011, the adjusted odds ratio to engage in WMC was 1.19 in 2014 (*P* = 0.05) and 1.32 in 2017–18 (*P* < 0.001), indicating an increase in the 2010s. WMC among CMWRAs who were using no contraceptive method slightly decreased from 58% in 2011 to 56% in 2017–18, while that among CMWRAs using traditional contraceptive methods increased from 63% to 72%. The determinants analysis found women’s working status, pregnancy status, desire for a child, and years of cohabitation as important predictors of WMC in CMWRAs.

**Conclusions:**

The increase in WMC may be due to increased openness in reporting sexual activity among women. Furthermore, working women engaged in higher WMC, which may be related to their active engagement in decision-making and increased sexual satisfaction.

Sexual intercourse is a key component of human reproduction, health, and overall well-being [1,2]. Sexual activity helps lower the heart rate and blood pressure by reducing stress and promoting oxytocin release [3–5]. Conversely, reduced sexual activity has been linked to higher mortality and poorer self-reported health [6,7]. However, these associations should be interpreted cautiously since sexual activity and health outcomes may share common underlying factors – for example, because healthier individuals likely engage more frequently in sexual activity.

Sexual intercourse is also an essential component of enjoying married life and a spousal bond [8,9]. Marriage in many societal contexts is considered the main dyadic relationship within which sexual activity occurs [10,11]. The frequency of sexual intercourse among married or cohabiting couples (also known as coitus) serves as an important component centering fertility and conjugal satisfaction [12,13]. Therefore, understanding the pattern of coitus is essential when evaluating the need for family planning services, sexual health, marital satisfaction, and social marital phenomena like divorce, extramarital affairs, and so on [14,15].

A couple’s pattern of coitus can be influenced by various biological, psychological, and socioeconomic factors [16,17]. For example, hormonal levels, age, and health status are key biological factors influencing sexual activity [18,19]. Higher levels of testosterone and oestrogen influence neurotransmitters such as dopamine, which are directly linked to pleasure and reward systems in the brain, thereby promoting sexual activity [20,21]. However, hormonal changes, especially during periods, pregnancy, and menopause in women can reduce sexual desire and function, resulting in less frequent coitus [22,23]. The pattern of coitus also changes with an individual’s age [16,24], while chronic illnesses like diabetes and cardiovascular disease can adversely affect sexual function [25].

Mental health disorders like depression, anxiety, and stress can lower sexual desire and reduce the frequency of coitus among couples due to decreased libido and energy levels [26,27]. Additionally, relationship satisfaction, characterized by emotional intimacy and effective communication, may often lead to more regular sexual activity among couples [28,29]. An individual’s perception of body image and appreciation from a partner may also impact sexual function; individuals with a positive perception may feel more confident to engage in sexual activity, while those with a negative perception may avoid it or are less likely to engage in sexual activity [30,31].

Changes in socioeconomic factors can also significantly influence the pattern of coitus over time [32]. Financial stability and independence can reduce stress, boost confidence, and create an engaging environment more conducive to frequent sexual activity, while financial stress and dependency may have the opposite effect [32,33]. Higher educational attainment facilitates better awareness regarding sexual health and contraception for an individual, which inspires the practice of more responsible sexual behaviour and enables informed family planning decision-making [29]. Additionally, cultural norms and societal values can shape sexual activity and affect sexual satisfaction [34]. Besides these couples’ age gap, parity, desire for a child, privacy, duration of the marriage, contraceptive use, religion, and other factors can also influence the frequency and pattern of their coitus [24,35].

While most existing studies on the changes in coitus, sexual intimacy, or sexual satisfaction between couples have taken place in developed countries, research in South Asia remains sparse, despite the region being home to nearly two-thirds of the world’s population and being characterised by unique sociocultural dynamics [36]. Specifically, only a few studies have assessed the pattern of coitus at the community level in India [36,37]. From a sociocultural context, South Asia holds comparatively conservative attitudes towards sexuality, traditional family structures, and gender equality, which may influence sexual behaviour and coitus [38,39]. Understanding coital frequency in this region is a crucial public health demand, as these specific sociocultural norms intersect with other reproductive health challenges, such as high rates of unintended pregnancies, limited access to modern contraception, and high fertility preferences [40].

Bangladesh, the third most populous country in South Asia, presents a compelling case for studying coitus [41]. Over the past decade, the predictors of coitus in the country have undergone notable changes, including progress in socioeconomic conditions, health behaviours, nutritional status, and contraceptive practices [42]. Despite these changes, there exists no empirical evidence of the pattern of coitus in Bangladesh, leaving a critical gap in our understanding of sexual behaviour in the country.

The total fertility rate (TFR) in Bangladesh has remained stagnant at 2.3 for the past 11 years [43]. However, one-third of the reproductive-age women did not use any method of contraception, while the prevalence of traditional methods ranged between 8% to 10% during this period [44–46]. Women using no or traditional contraceptive methods are at higher risk of unintended pregnancies and abortions compared to those women who are using any modern method [47]. In fact, 0.27 million unwanted births, 0.43 million mistimed pregnancies, and 1.2 million abortions occur in Bangladesh annually [46,48,49]. Studying the pattern of coitus among women with no method or using traditional methods is therefore crucial for understanding future threats of unintended pregnancies.

Despite this, the patterns of coitus and other sexual behaviours remain almost unstudied in Bangladesh. In 1982, Ruzicka and Bhatia analysed the behaviour of sexual intercourse between married women aged under 50 years in 22 villages of the International Centre for Diarrhoeal Disease Research, Bangladesh (icddr,b) field operation area in the Matlab sub-district in Bangladesh [50]. In agreement with studies in other contexts, Ruzicka and Bhatia also found that young, newly married, non-breastfeeding, non-contraceptive users, and pregnant women have a higher coital frequency. However, the credibility of their findings was questionable due to a high non-response (one-third) to the sexual intercourse question due to the social sensitivity of the questions.

No other study apart from this one by Ruzicka and Bhatia has examined any population-representative aspects of coitus in Bangladesh, particularly at the national level. Therefore, we aimed to investigate temporal changes in the pattern of weekly marital coitus (WMC), defined as whether a woman reported engaging in sexual activity within the week preceding the survey, and the associated predictors of WMC among currently married women of reproductive age (CMWRA), *i.e.* women aged 15–49 years in Bangladesh.

We, therefore, had three objectives: to analyse the changes in marital coitus in Bangladesh in the 2010s; to analyse the changes in marital coitus among CWMRAs with no or traditional contraceptive methods in Bangladesh in the 2010s; to identify the sociodemographic predictors of marital coitus among CMWRAs in Bangladesh.

## METHODS

### Data

We combined data from three rounds of the Bangladesh Demographic and Health Surveys (BDHS) conducted in 2011, 2014, and 2017–18 to analyse the changes in the WMC among CMWRA overall and in subgroups who were using either no contraceptive methods or traditional contraceptive methods across these years. We further examined the sociodemographic and health factors associated with WMC in this population.

**Figure 1:**
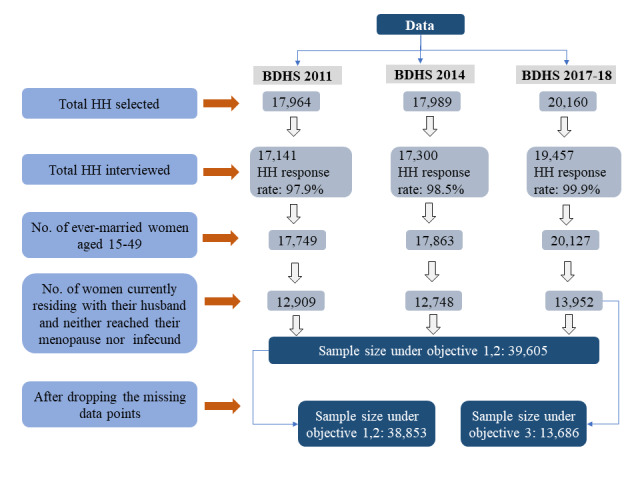
Flow diagram of the data used in this study

The BDHSs are nationally representative household surveys that interview ever-married women of reproductive age only, which allows for the study of the population’s demographics and health characteristics, including their marriage and sexual activity, fertility, child mortality, reproductive behaviour, and so on. All surveys adopted a two-stage stratified cluster sampling design (**Figure 1**), details of which can be found elsewhere [44–46].

Social norms and religions in Bangladesh do not allow sexual intercourse outside of wedlock, which is why we limited our analyses to marital coitus only. As patterns of coitus among women whose husbands were living elsewhere may differ and be less regular compared to those of women whose husbands were living with them, we included only the former in our study. Additionally, infecund women and those who have reached menopause typically experience a reduction in sexual frequency due to physiological changes (*i.e.* decline in oestrogen and progesterone levels in women) and reduced reproductive motives, which may impact regular marital coitus patterns [51,52]. We thus also excluded these groups, as their inclusion could have introduced variability that may have influenced patterns and determinants of marital coitus among women still in their reproductive years.

### Outcome measure

The main outcome of interest was whether the couple was engaged in coitus in the last week preceding the survey. The BDHSs included questions about a currently married couple's recent sexual activity from the 2011 round: ‘Was the CMWRA involved in sexual activity in the last four weeks preceding the survey?’. Based on the response to this question from BDHSs, we constructed the dichotomous outcome variable, where ‘yes’ indicated that the CMWRA has had sex in the past seven days preceding the survey and ‘no’ indicated otherwise.

The rationale for choosing a week is that weekly sexual intercourse captures the most prevalent sexual behaviour patterns among married or cohabiting couples worldwide [53,54]. Additionally, measuring coital frequency weekly helps capture variations in coitus patterns and maintain recall accuracy, unlike measuring longer intervals (*e.g.* monthly) that may miss subtle variations across different characteristics of couples [55,56].

### Covariates of interest

Previous studies of sexual activity [35,50,57,58] and conceptual understanding suggest several characteristics of the couple may predict the regularity of coitus. For better understanding, we have grouped our covariates of interest into five groups: time, demographic characteristics, socioeconomi characteristics, family planning and health status, and geographical location (**Table 1**).

**Table 1:** The list of covariates of interest under each section

Sections	Covariates of interest
Time	Interview year (used as categorical data); interview month.
Demographic characteristics	Woman’s age (numeric data recoded to categorical); husband’s age (numeric data recoded to categorical); marriage duration (numeric data recoded to categorical); number of living children (numeric data recoded to categorical); husband-wife age gap (numeric data recoded to categorical), and religion.
Socio-economic characteristics	Women’s education (numeric data recoded to categorical); husband’s education (numeric data recoded to categorical); women’s working status; wealth quintile; urban or rural residence; privacy level of the household (number of members living per room).
Family planning, health status, and desire for a child	Body mass index (weight/height^2^) of women (<18.5: underweight, 18.5 to <25.0: normal, 25.0 to <30.0: overweight, ≥30.0: obese); current pregnancy status; family planning method (categorical; long-acting reversible and permanent methods: intrauterine device, implant, tubal ligation, no-scalpel vasectomy, short-acting methods: injectables, pill, and condom, traditional: all other methods and no methods); desire for child.
Geographical location	Region (west: Khulna, Rajshahi, and Rangpur divisions, central: Dhaka, Mymensingh, and Barishal divisions, and eastern: Chattogram and Sylhet divisions).

#### Time

Coital frequency or behaviour in a population can vary annually or seasonally [59]. Temporal factors, such as the year and month of the interview, can help to capture seasonal or annual shifts in coital frequency. These shifts may happen due to changes in weather, social norms, health care access, or cultural attitudes over time, which make ‘time’ an important factor for understanding trends in coitus throughout the study period.

#### Demographic characteristics

Demographic characteristics of a couple, like age, marriage duration, number of children, husband-wife age gap, and religion, are associated with marital relationships, sexual engagement, and fertility intentions [16,60]. They are thus foundational to understanding the dynamics of coital behaviour, as they directly influence fertility motivations and relationship dynamics.

#### Socioeconomic characteristics

Factors like education, working status, wealth, urban or rural residence, and household privacy shape individuals’ lifestyles, autonomy, and quality of life [16,32,61]. They often affect a couple’s sexual health awareness and openness in communication, impacting intimacy. A household’s privacy level is also connected with the couple’s ability to engage in sexual activity.

#### Health status and family planning

Health indicators, including body mass index (BMI), pregnancy status, and family planning method, can directly influence a couple’s physical readiness and sexual engagement [62]. The desire for children can significantly affect coital frequency, especially in the context of fertility motivations.

#### Geographical location

Regional differences within Bangladesh (west, central, and eastern divisions) can also introduce potential variations for analyses such as ours due to cultural, socioeconomic, and healthcare access disparities across regions, all of which can shape marital intimacy and frequency.

### Statistical analysis

To gain better insight into the data, we first conducted a univariate analysis of the selected predictors of WMC. Then, we estimated the prevalence of CMWRAs engaged in WMC across their different characteristics. We used the survey weight-adjusted Rao-Scott χ^2^ test of association to explore the association of predictors with WMC.

To examine the factors associated with WMC, we employed mixed-effect logistic regression analysis, the most widely used regression framework in epidemiological studies with binary outcomes of interest. Conventional multivariable logistic regression can capture the fixed effects only. Thus, in the presence of cluster-level variation in the outcome of interest, using a fixed-effect logistic regression model can bias the estimates and, consequently, the standard error. The nested structure of BDHS data (women nested within cluster-primary sampling unit) often creates cluster-level variation in the outcome of interest. Therefore, we first constructed the null model with random intercepts at the cluster level to estimate the degree of correlation in WMC at the cluster level. To further examine the trend, we added all the mentioned covariates in a mixed-effect logistic regression model considering random intercept at the cluster level. We used the same model specification to identify the correlates. Using this mixed-effect logistic regression model while considering random intercept at the cluster level helped deal with the hierarchical nature of the clustered data by incorporating both fixed effects and random effects at the cluster level. The former represents the average relationship between the predictors and the response variable across all clusters, while the latter captures the variation across clusters by explicitly modeling the clustering structure.

All analyses incorporated the survey weights (Table S1 in the **Online Supplementary Document**). The three BDHSs were conducted at different time points, and each survey had separate normalised survey weights. Since we pooled survey data sets from three different time points, we denormalised the survey weights to adjust for the sample fraction of the population of that year.

## RESULTS

### Univariate analysis of the study sample

**Table 2** shows the background characteristics of CMWRAs in the three BDHS rounds (2011, 2014, and 2017–18), revealing gradual demographic and socioeconomic shifts. Over time, there was an increase in urban residency and a significant rise in women's participation in the workforce. Educational attainment also improved among both women and their husbands. The BMI declined in underweight women and rose in overweight and obese women. Contraceptive use remained dominated by short-acting methods across all years, though a modest increase in traditional and long-acting method use was noted. The desire for more children remained stable, with approximately two-thirds of women reporting they did not want more children.

**Table 2:** Univariate characteristics for all the covariates of the study sample for all three BDHSs, presented as n (%)

	BDHS 2011	BDHS 2014	BDHS 2017–18
**Total**	12 536 (100)	12 622 (100)	13 686 (100)
**Age of women in years**			
15–19	1496 (12.2)	1578 (12.4)	1408 (10.7)
20–24	2687 (21.7)	2470 (19.9)	2520 (18.3)
25–34	4687 (37.1)	4969 (39.7)	5286 (38.6)
35–49	3666 (29)	3605 (28)	4472 (32.4)
**Husband's age in years**			
≤24	762 (6.4)	792 (6.1)	746 (5.7)
25–34	3936 (31.7)	3975 (32)	4001 (29.5)
35–49	5598 (44.1)	5596 (44.2)	6428 (46.4)
≥50	2240 (17.8)	2259 (17.7)	2511 (18.4)
**Years since first cohabitation**			
<3	1424 (11.4)	1558 (12.3)	1462 (10.7)
3–9	3490 (27.6)	3359 (26.9)	3604 (26.2)
10–19	4339 (34.5)	4468 (35.5)	4774 (34.7)
≥20	3283 (26.4)	3237 (25.3)	3846 (28.4)
**Parity**			
No child	1060 (8.6)	1159 (8.8)	1183 (8.8)
Two children	6686 (52.5)	7002 (55.4)	7663 (55.5)
Three or more children	4790 (39)	4461 (35.8)	4840 (35.7)
**Age gap**			
0–9 years	7481 (59.9)	7988 (63)	9179 (67.2)
10–19 years	4555 (36.1)	4092 (32.5)	4023 (29.3)
≥20 years	500 (3.9)	542 (4.5)	484 (3.5)
**Religion**			
Muslim	11 034 (89.4)	11 303 (89.4)	12 177 (89.5)
Non-Muslim	1502 (10.6)	1319 (10.6)	1509 (10.5)
**Women's education level**			
No	5373 (45)	4922 (40.8)	4769 (35.8)
Primary	1506 (12)	1487 (11.3)	1506 (10.7)
Less than secondary	3987 (31.3)	4227 (33.3)	4921 (37)
Secondary or higher	1670 (11.7)	1986 (14.5)	2490 (16.6)
**Husband's education level**			
No	5800 (48.5)	5619 (45.4)	5729 (43)
Primary	1458 (11.5)	1480 (11.6)	1696 (12.5)
Less than secondary	2728 (21.4)	2834 (22.8)	3264 (24.1)
Secondary or higher	2550 (18.6)	2689 (20.3)	2997 (20.4)
**Women's working status**			
No	11 032 (88.2)	8667 (67)	6894 (49.9)
Yes	1504 (11.8)	3955 (33)	6792 (50.1)
**Asset quintile**			
Poorest	2306 (19.5)	2461 (19.9)	2787 (20)
Poorer	2417 (20.3)	2445 (19.5)	2680 (20.1)
Middle	2419 (20.3)	2485 (19.6)	2563 (19.5)
Richer	2569 (19.8)	2559 (20.1)	2747 (20.7)
Richest	2825 (20.2)	2672 (20.9)	2909 (19.7)
**Residence**			
Rural	8098 (73.7)	8175 (70.9)	8468 (70)
Urban	4438 (26.3)	4447 (29.1)	5218 (30)
**Person per room**			
<2	4465 (33.6)	5027 (38.5)	5893 (42.2)
2–4	6400 (52.1)	6236 (50)	6461 (47.9)
≥5	1671 (14.4)	1359 (11.5)	1332 (9.9)
**BMI**			
Normal	7524 (61.1)	7298 (58.7)	7597 (56.1)
Under-weight	2799 (22.7)	2209 (17.2)	1581 (11.2)
Over-weight	1777 (13.1)	2472 (19)	3464 (25.1)
Obese	436 (3.1)	643 (5.1)	1044 (7.6)
**Pregnancy status**			
No/unsure	11 629 (92.8)	11 707 (92.8)	12 770 (93.3)
First trimester	240 (1.9)	239 (1.8)	262 (1.8)
Second trimester	357 (2.8)	366 (3)	370 (2.7)
Third trimester	310 (2.4)	310 (2.4)	284 (2.2)
**Current contraceptive method**			
Not using	3060 (24.4)	2782 (22)	2807 (20.5)
Short-acting	6812 (54.3)	7240 (57.4)	7616 (55.6)
Traditional	1368 (10.9)	1242 (9.8)	1763 (12.9)
Long-acting	1296 (10.3)	1358 (10.8)	1500 (11)
**Desire for more child**			
No more	7626 (66.6)	7347 (65.4)	8002 (64.5)
Want more	3790 (33.4)	3928 (34.6)	4365 (35.5)
**Region**			
West	5875 (41.8)	5743 (35.7)	5284 (35.1)
Central	3529 (37.7)	3645 (41.5)	4854 (36.6)
East	3132 (20.5)	3234 (22.8)	3548 (28.3)

BDHS – Bangladesh Demographic and Health Survey, BMI – body mass index

### Changes in weekly coitus pattern during the 2010s

#### Bivariate analysis on the pooled dataset

We found that the percentage of CMWRAs who had intercourse in the week preceding the survey increased with time. Weekly intercourse among CMWRAs was reported by 66% of CMWRAs in 2011, 71% in 2014, and 76% in 2017–18, indicating a rising trend in WMC over time (Table S2 in the **Online Supplementary Document**).

#### Mixed-effect logistics regression on the pooled dataset

The patterns of WMC among CMWRAs between 2011 and 2017–18 showed an upward trend. In comparison to 2011, according to the adjusted odds ratio, CMWRAs were 19% more likely to engage in WMC in 2014 compared to 32% in 2017–18 (Table S3 in the **Online Supplementary Document**).

### Changes in the WMC among CMWRAs using no or traditional methods during the 2010s

**Figure 2:**
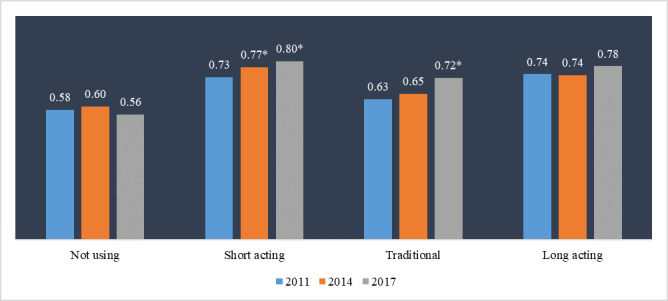
Marginal probabilities of the WMC among CMWRAs: estimated from model 2. *P < 0.001.

The marginal probabilities of women not using any contraceptives engaging in weekly coitus showed a slight fluctuation, increasing minimally from 0.58 in 2011 to 0.60 in 2014, but then decreasing to 0.56 in 2017. In contrast, among women using traditional contraceptive methods, there was a steady increase in marginal probabilities of engaging in weekly coitus, rising from 0.63 in 2011 to 0.65 in 2014, and then further to 0.72 in 2017 (*P* < 0.001) (**Figure 2**).

### WMC among CMWRAs and their predictors

#### Bivariate analysis

Having sexual intercourse in a week was prevalent in 75.8% of CMWRAs who are currently living with their husbands (Table S4 in the **Online Supplementary Document**).

In detail, WMC was lower among older couples with high parity and more age gap. For example, 78% of women aged <20 years reported that they engaged in WMC compared to 70% of women aged 35–49 years. Further, 78% of women reported doing so if the husband was aged <24 years, compared to 64% if he was >50 years old. Moreover, WMC was 79% among the couples with no child and 72% who had three or more; 78% if the couple's age difference was less than 10 years; and 66% if the age difference was >20 years. Also, WMC was lower among women who were less educated and higher among working-class women. Couples of the richest economic classes and those who get more privacy in their household had higher percentages of WMC. Couples using short-acting family-planning methods had a higher frequency of WMC (82%) compared to those not using any family-planning method (64%).

#### Mixed-effect logistics regression

After controlling for the background characteristics, the mixed-effects logistic regression model showed that the couple's age, religion, women’s educational attainment and nutritional status, residence type, and regional variation were independent of the WMC pattern (**Table 3**).

**Table 3:** Mixed effect logistic regression of WMC by different characteristics of CMWRAs using BDHS 2017–18

	aOR (95% CI)	*P*-value
**Age of women in years**		
15–19	ref	
20–24	1.08 (0.85,1.36)	0.529
25–34	1.23 (0.94,1.61)	0.137
35–49	1.12 (0.78,1.60)	0.533
**Husband's age in years**		
≤24	ref	
25–34	1.2 (0.94,1.53)	0.153
35–49	1.23 (0.94,1.61)	0.133
≥50	0.9 (0.64,1.26)	0.527
**Years since first cohabitation**		
<3	ref	
3–9	1.45 (1.15,1.84)	0.002
10–19	1.44 (1.08,1.93)	0.014
≥20	1.08 (0.75,1.55)	0.693
**Parity**		
No child	ref	
Two children	0.5 (0.39,0.64)	<0.001
Three or more children	0.5 (0.38,0.67)	<0.001
**Age gap**		
0–9 years	ref	
10–19 years	0.78 (0.70,0.88)	<0.001
≥20 years	0.77 (0.59,1.01)	0.062
**Religion**		
Muslim	ref	
Non-Muslim	1.02 (0.85,1.21)	0.861
**Women's education level**		
No	ref	
Primary	1.1 (0.92,1.32)	0.284
Less than secondary	1.02 (0.89,1.17)	0.792
Secondary or higher	0.97 (0.79,1.19)	0.764
**Husband's education level**		
No	ref	
Primary	0.93 (0.80,1.09)	0.398
Less than secondary	1.01 (0.88,1.16)	0.886
Secondary or higher	0.79 (0.67,0.94)	0.007
**Women's working status**		
No	ref	
Yes	1.24 (1.11,1.39)	<0.001
**Asset quintile**		
Poorest	ref	
Poorer	1.11 (0.95,1.30)	0.184
Middle	0.94 (0.79,1.11)	0.451
Richer	1.14 (0.95,1.37)	0.162
Richest	1.43 (1.15,1.78)	0.001
**Residence**		
Rural	ref	
Urban	0.96 (0.84,1.10)	0.574
**Person per room**		
<2	ref	
2–4	0.98 (0.88,1.09)	0.665
≥5	0.81 (0.68,0.96)	0.017
**BMI**		
Normal	ref	
Underweight	1.01 (0.86,1.18)	0.89
Overweight	1.06 (0.94,1.19)	0.333
Obese	1 (0.83,1.20)	0.986
**Pregnancy status**		
No/unsure	ref	
First trimester	3.72 (2.34,5.91)	<0.001
Second trimester	1.32 (0.95,1.82)	0.101
Third trimester	0.4 (0.29,0.55)	<0.001
**Current contraceptive method**		
Not using	ref	
Short-acting	3.6 (3.15,4.12)	<0.001
Traditional	2.31 (1.92,2.78)	<0.001
Long-acting	3.13 (2.38,4.11)	<0.001
**Desire for more child**		
No more	ref	
Want more	1.74 (1.48,2.05)	<0.001
**Region**		
West	ref	
Central	1.08 (0.94,1.23)	0.284
East	1.09 (0.94,1.26)	0.236

aOR – adjusted odds ratio, BMI – body mass index, CI – confidence interval, ref – reference

Couples who have been living together for 3–9 years were 45% more likely to engage in WMC than those who had been cohabiting for <3 years. The chances of engaging in WMC were half among couples with three or more children compared to those who had no children. Working women were 24% more likely to have had weekly intercourse than non-working women. Women whose husbands completed secondary school or above were 21% less likely to engage in WMC compared to women whose husbands never attended school. The richest couples were 43% more likely to engage in WMC compared with the poorest couples. When the number of people living per room was more than five, the chance of having weekly intercourse was 19% less likely compared to households where fewer than two people were living per room. Women in the first trimester of pregnancy were more than three times more likely to engage in WMC than women who were not pregnant. Couples using short-acting family-planning methods exhibited more than three times higher chances of engaging in WMC compared to those not using any family-planning method.

## DISCUSSION

In this study, we aimed to assess the changes in WMC patterns in CMWRAs in Bangladesh from 2011 to 2017–18, with a focus on understanding whether there has been an increase in WMC among women using no contraceptive methods or traditional methods, which could give us a sense of future risk of unintended pregnancies. Additionally, we explored the key predictors influencing WMC in the Bangladeshi context.

### Changes in weekly coitus pattern over time

From 2011 to 2017, we found an increase in the proportion of CMWRA having weekly intercourse. This upward trend in WMC can be linked to several factors, which we outline below.

### Changing societal attitudes and norms

Between 2011 and 2018, Bangladesh underwent substantial social changes, including a rise in women’s educational attainment (from 72% in 2011 to 83% in 2017–2018), universal access to mobile and internet (above 60%), an increase in the working-class women (from 13% in 2011 to 48% in 2017–2018), and progressive shifts in women’s empowerment [44,46,63]. These developments may have contributed to reducing the stigma associated with discussing sexual topics, even within survey contexts. Additionally, increased awareness of sexual and reproductive health – promoted by government initiatives and non-governmental organisation programmes – may encourage women to adopt more egalitarian views, making them more comfortable with openly reporting sexual behaviours [64].

### Unmet contraceptive needs

Higher unmet needs are connected with decreased coital frequency, as couples will revert to intramarital abstinence as a reliable substitute for contraception [65]. A decrease in unmet contraception needs in Bangladesh (14% in 2011 to 12% in 2017) and a desire for birth spacing may have contributed to the rise in WMC [44,46,66].

### Economic stability, privacy, and nutritional improvement

National economic stability facilitates people to have greater access to contraceptives, better housing, and more privacy, all of which can lead to an increase in marital sexual activity [67], as has been the case with Bangladesh’s significant economic growth over the past ten years [44,46]. We found that couples with greater privacy and those in the richest quintile have more regular coitus. Studies regarding sexual relationships and satisfaction in countries like India also support these findings [36]. Nutritional improvement over time can lead to increased sexual activity between married couples for a variety of reasons, including higher energy, improved physical health, hormonal balance, and increased confidence and stress reduction [68]. Bangladesh has made significant progress in improving nutritional conditions across all age groups, which may have contributed to improved sexual relations among married couples over time [69].

### Coitus pattern among CMWRAs with no/traditional method and policy suggestion

Our study showed a significant increase in WMC among CMWRAs using traditional contraceptive methods from 2011 to 2017–18, which raised concerns about a potential rise in unintended pregnancies in the future. Couples who use natural family planning or traditional methods, such as periodic abstinence or withdrawal, often require joint fertility tracking, more open discussion regarding sexual engagement, and joint family planning decisions, which may improve intimacy that positively influences their coital frequency [70]. Also, greater accessibility and increased sexual satisfaction associated with traditional contraceptive methods may contribute to higher coital frequency among couples using these methods [71,72].

The family planning programmes in Bangladesh successfully increased the contraceptive prevalence rate by converting women using no contraceptive methods to modern methods [44–46]. However, data from 1996 to 2017 showed a persistent prevalence (8% to 10%) of traditional family planning methods, indicating that transitioning women from traditional family planning methods through government family planning programmes to modern methods remained less effective. Couples often use traditional methods due to concerns over the side effects of modern methods, lack of awareness, or misconceptions [73]. To address this gap, the government should strengthen family planning strategies to actively engage couples who rely on traditional methods and thus encourage a shift toward modern methods. This could include targeted awareness campaigns through mass media and the distribution of leaflets that address specific concerns about the side effects of modern contraceptives, increase awareness of the efficacy of modern methods, and dispel misconceptions. Additionally, effective family planning counseling in antenatal care visits could address individual health concerns and preferences, enabling more informed decisions around contraceptive use. Since nearly one-third of currently married women of reproductive age still use no contraceptive method, it is critical to enhance efforts to bring them under the umbrella of modern family planning methods to reduce the risk of unintended pregnancies and ensure better reproductive health outcomes.

### Key predictors of marital coitus in Bangladesh

According to our findings, engagement in sexual activity among both men and women does not show significant variation across the age span of 15–49 years. We found WMC increased as time went on compared to the beginning of the cohabitation, and then began to decline after the cohabitation length exceeded 20 years. According to Ovid and colleagues [60], the length of marriage should increase in coital frequency (an experience effect in marriage) if practice improves sexual skill and sexual skill improves sexual pleasure.

Our findings suggest that, during the first trimester of pregnancy, CMWRA experience an increased WMC compared to CMWRA who are not pregnant, while in the third trimester, there is a significant decrease in coitus among married women. Psychologically, couples might experience heightened intimacy during early pregnancy, as the news of conception can bring excitement and a stronger sense of connection, fostering more frequent intimacy. Further, hormonal changes (*i.e.* increased levels of oestrogen and progesterone) during the first trimester may increase women’s libido, resulting in more intimacy than regular [74]. In addition, the intention to conceive followed by a delay in conception detection (particularly in Bangladesh) may increase coitus during the early stage of the first trimester. Moreover, cultural and religious norms in Bangladesh do not discourage sexual activity during the early stage of pregnancy. In contrast, due to physical and psychological discomfort, decreased libido, exhaustion, nausea, vomiting, increased frequency and urgency of urination, and fear of harming the fetus in the third trimester decreases the frequency of coitus significantly [62,75]. Decreased sexual activity among pregnant women in the third trimester was observed in other Asian countries like China [76].

We found that the number of children has a negative relationship with WMC. Couples with fewer or no children may have greater freedom and privacy, which can provide a more conducive space to be sexually engaged. According to our study, WMC patterns remained similar across educational groups of CMWRA, but husbands with higher educational attainment had coitus less frequently. In Bangladesh's social context, husbands with higher education often bear greater professional responsibilities and face heightened financial pressures to maintain or elevate their family’s social standing and expectations [77]. These demands can lead to extended work hours, increased stress, and physical exhaustion, which may, in turn, limit their energy levels and availability for sexual engagement [78].

Working-class women engaged in greater sexual activity than nonworking women did, according to our study. For working women in Bangladesh, financial independence and employment could contribute to a sense of self-worth and mutual respect within the marriage [79]. This aligns with social exchange theory, which suggests that relationships are built on the costs and benefits exchanged between partners [80,81]. Employed women may feel a stronger sense of personal agency, well-being, and attractiveness, and they may reduce economic dependence on their spouses, which can positively influence relationship dynamics, including sexual engagement [82,83].

According to our research, predictors like different regional areas, religion, and residency type were not related to WMC among CMWRAs.

### Limitations

Several limitations to this study should be mentioned. The accuracy of self-reported data on WMC is dependent on participants' capacity and desire to accurately recall and record their sexual activity, which can introduce measurement errors [14]. Cultural norms and societal stigmas associated with discussing sexual matters may influence participants' willingness to divulge information about their sexual conduct, potentially leading to under- or over-reporting. Additionally, as they can vary across the sociodemographic contexts, participants’ willingness to respond about their sexual activity can be affected differently, which eventually may result in either underreporting or overreporting.

Ensuring full privacy during interviews often becomes challenging in survey [55]. This may make the respondents discomfort in responding to sensitive questions like sexual activity, which may result in potential underreporting [55]. To ensure full privacy during the interviews, we suggest building a strong rapport with household members. We also recommend informing the respondent about the importance (*i.e.* understanding marital relationships, fertility, family planning, *etc*.) of accurate reporting on coitus.

## CONCLUSIONS

There has been no research done on the pattern of marital coitus in Bangladesh at the national level. From a sociocultural and historical context, the country holds comparatively less egalitarian attitudes toward sexuality, which may have shaped sexual behaviour and activity differently. However, the increase in WMC from 2011 to 2017–18 we observed here may suggest a shift in cultural norms and a greater openness among women in reporting their sexual activities. Higher WMC among working-class women might be a reflection of greater autonomy in women’s decision-making. The increase in WMC among women using traditional contraceptive methods may indicate a potential risk of unintended pregnancies, which underlines the need for more effective family planning strategies and awareness programmes to promote the use of modern contraceptive methods. Future research should focus on in-depth understanding of the underlying reasons for these changes and the long-term impacts on reproductive health outcomes.

## Additional material


Online Supplementary Document
