# Supplementary material for: Marital coitus in Bangladesh in the 2010s: trends and sociodemographic determinants
Source: J Glob Health. 2025 Jun 27;15:04139. doi: 10.7189/jogh.15.04139 (PMC12201935; doi:10.7189/jogh.15.04139)
Supplement: Online Supplementary Document [file jogh-15-04139-s001.pdf]

Supplement to: Amin MT, Rahman MM, Khan SH, Bhuiyan A, Rahman M, Alam N, Haider MM. Marital coitus in Bangladesh in the 2010s: trends and sociodemographic determinants. J Glob Health. 2025;15:04139.

Table S1. Adjustment of survey weights for pooled data analysis of the 2011, 2014, and 2017–18 BDHS

Index(i)	BDHS year	Total population in the country (million) [a]	Proportion of ever married women ages 15–49 years in the population [b]	Total ever-married women in the country (million)	Ever-married women interviewed	Proportion of women interviewed	Women to be interviewed in survey i to have same proportion of women interviewed in all surveys (Base year 2017–18)	Factors to be multiplied by weights for ever-married women respondents
(1)	(2)	(3)	(4)	(5) =(3)×(4)	(6)	(7) =(6)/[(5) × 1000000]	(8) =[(7)1/ (7)i]×(6)i	(9) =(8)/(6)
1	2017–2018	161	0.24	38.64	20127	0.000521	20127	1.000000
2	2014	155	0.24	37.20	17863	0.000480	19377	1.084752
3	2011	149	0.23	34.27	17749	0.000518	17851	1.005732

[a] Source: United Nations World Population Prospects (https://population.un.org/wpp/Download/Standard/Population/). Access date: 4 June 2021. Population average of two years is used where the survey spanned over two calendar years.
[b] Source: Bangladesh DHS (https://dhsprogram.com/). Estimated as [(# of ever-married women ages 15–49 years interviewed) ÷ (# of household members recorded)].

Table S2. Percentage of CMWRAs who engaged in WMC using the pooled dataset:

Explanatory variables	% of CMWRAs had WMC	Number	Chi-square value
**Total**	**70.9**	**28,527**	
**Year**			
2011	65.9	8,385	101.6***
2014	70.8	9,858	
2017-18	75.8	10,284	
**Age of women**			
15-19	74	3,510	104.9***
20-24	74.4	5,963	
25-34	74.2	11,479	
35-49	63.1	7,575	
**Husband's age**			
24<=	75.6	1,836	158.2***

Explanatory variables	% of CMWRAs had WMC	Number	Chi-square value
25-34	74.8	9,346	
35-49	72.9	13,165	
50+	57.8	4,182	
**Years since fisrt cohabitation**			
<3	73.3	3,383	128.2***
3 to 9	74.6	8,077	
10 to 19	74.3	10,441	
20+	61.7	6,627	
**Parity**			
No child	76.9	2,697	82***
2 child	73	16,013	
3+ child	66.4	9,818	
**Age gap**			
0-9 years	73	18,633	61.1***
10 to 19 years	68.4	8,955	
20+ years	58.3	939	
**Religion**			
Muslim	71.2	25,611	9.1**
Non-Muslim	68.6	2,916	
**Women's education level**			
No	66.9	10,896	50.2***
Primary	72.3	3,289	
<Secondary	73.6	10,030	
>=Secondary	74.8	4,313	
**Husband's education level**			
No	69.6	12,760	6.7**
Primary	71.4	3,413	
<Secondary	72.7	6,667	
>=Secondary	71.5	5,688	
**Women's working status**			
No	69.2	18,929	78.5***
Yes	74.5	9,598	
**Asset quintile**			
Poorest	69.1	5,506	15.7***
Poorer	70.2	5,637	
Middle	68.7	5,467	
Richer	71.6	5,810	
Richest	74.8	6,108	
**Residence**			
Rural	69.7	20,045	52.9***
Urban	73.9	8,482	
**Person per room**			
<=2	71	10,899	13.6***
2 to 4	71.8	14,424	
5+	67	3,204	
**BMI**			
Normal	70.8	16,697	3.1*
Under-weight	69.5	4,731	
Over-weight	72.3	5,575	
Obese	71.4	1,524	
**Pregnancy status**			
No/Unsure	71.6	26,778	78.2***
1st trimster	80	589	
2nd trimster	64.6	742	
3rd trimster	44.4	419	
**Current contraceptive method**			
Not using	61.7	5,589	196.9***
Short-acting	76.9	17,182	

Explanatory variables	% of CMWRAs had WMC	Number	Chi-square value
Tradiional	62.7	2,819	
Long-acting	67.9	2,937	
**Desire for more child**			
No more	68.2	16,211	210.4***
Want more	77.3	9,681	
**Region**			
West	69.7	10,493	7.4**
Central	72.2	11,223	
East	70.7	6,811	

Note:* $p < 0.05$, ** $p < 0.01$,***$p<0.001$

Table S3. Adjusted odds ratio, from mixed-effect logistic regression, of WMC by different characteristics of CMWRAs, using pooled data

	Model 1		Model 2	
Explanatory variables	Adjusted odds ratio	Confidence Interval	Adjusted odds ratio	Confidence Interval
**Year**				
2011	Reference		Reference	
2014	1.19**	[1.09,1.30]	1.07	[0.92,1.24]
2017-18	1.32**	[1.19,1.46]	0.91	[0.78,1.05]
**Current contraceptive method**				
Not using	Reference		Reference	
Short-acting	2.51**	[2.31,2.73]	2.03**	[1.80,2.30]
Tradiional	1.49**	[1.32,1.70]	1.24**	[1.06,1.46]
Long-acting	2.28**	[1.94,2.68]	2.18**	[1.66,2.86]
**Interaction between current contraceptive method and the survey years**				
Short-acting, BDHS 2014	-	-	1.21*	[1.00,1.46]
Short-acting, BDHS 2017-18	-	-	1.67**	[1.43,1.95]
Traditional, BDHS 2014	-	-	1.04	[0.80,1.35]
Traditional, BDHS 2017-18	-	-	1.75**	[1.41,2.18]
Long-acting, BDHS 2014	-	-	0.91	[0.62,1.33]
Long-acting, BDHS 2017-18	-	-	1.36	[0.94,1.96]
**Age of women**				
15-19	Reference		Reference	
20-24	1.1	[0.94,1.29]	1.11	[0.95,1.31]
25-34	1.08	[0.91,1.28]	1.09	[0.92,1.30]
35-49	1.02	[0.81,1.29]	1.03	[0.82,1.29]
**Husband's age**				
24<=	Reference		Reference	
25-34	0.99	[0.85,1.14]	0.98	[0.85,1.14]
35-49	0.93	[0.79,1.11]	0.93	[0.79,1.11]
50+	0.65**	[0.53,0.81]	0.65**	[0.53,0.81]
**Years since fisrt cohabitation**				
<3	Reference		Reference	
3 to 9	1.38**	[1.19,1.60]	1.39**	[1.20,1.61]
10 to 19	1.61**	[1.36,1.92]	1.64**	[1.38,1.95]
20+	1.19	[0.96,1.48]	1.2	[0.97,1.49]
**Parity**				
No child	Reference		Reference	
2 child	0.52**	[0.45,0.59]	0.51**	[0.45,0.59]
3+ child	0.50**	[0.42,0.58]	0.49**	[0.42,0.58]

Explanatory variables	Model 1 Adjusted odds ratio	Model 1 Confidence Interval	Model 2 Adjusted odds ratio	Model 2 Confidence Interval
**Age gap**				
0-9 years	Reference		Reference	
10 to 19 years	0.96	[0.89,1.04]	0.97	[0.90,1.04]
20+ years	0.80**	[0.68,0.93]	0.80**	[0.69,0.94]
**Religion**				
Muslim	Reference		Reference	
Non-Muslim	0.91	[0.83,1.01]	0.91	[0.83,1.01]
**Women's education level**				
No	Reference		Reference	
Primary	1.11*	[1.01,1.23]	1.11*	[1.00,1.22]
<Secondary	1.03	[0.95,1.12]	1.03	[0.95,1.12]
>=Secondary	1.03	[0.91,1.17]	1.04	[0.92,1.18]
**Husband's education level**				
No	Reference		Reference	
Primary	0.95	[0.87,1.05]	0.96	[0.87,1.05]
<Secondary	0.95	[0.88,1.04]	0.96	[0.88,1.05]
>=Secondary	0.81**	[0.73,0.90]	0.81**	[0.73,0.90]
**Women's working status**				
No	Reference		Reference	
Yes	1.17**	[1.09,1.26]	1.16**	[1.08,1.25]
**Asset quintile**				
Poorest	Reference		Reference	
Poorer	1.07	[0.98,1.18]	1.08	[0.98,1.18]
Middle	1.01	[0.92,1.11]	1.01	[0.92,1.12]
Richer	1.13*	[1.01,1.25]	1.13*	[1.02,1.25]
Richest	1.31**	[1.15,1.49]	1.31**	[1.15,1.49]
**Residence**				
Rural	Reference		Reference	
Urban	1.01	[0.93,1.09]	1.01	[0.93,1.09]
**Person per room**				
<=2	Reference		Reference	
2 to 4	0.96	[0.89,1.02]	0.96	[0.89,1.02]
5+	0.80**	[0.72,0.89]	0.80**	[0.72,0.89]
**BMI**				
Normal	Reference		Reference	
Under-weight	0.99	[0.91,1.08]	0.99	[0.91,1.08]
Over-weight	1.04	[0.96,1.13]	1.04	[0.95,1.13]
Obese	1	[0.87,1.14]	1	[0.87,1.14]
**Pregnancy status**				
No/Unsure	Reference		Reference	
1st trimster	2.19**	[1.72,2.79]	2.20**	[1.73,2.80]
2nd trimster	1.04	[0.81,1.32]	1.04	[0.82,1.32]
3rd trimster	0.41**	[0.34,0.50]	0.42**	[0.34,0.51]
**Desire for more child**				
No more	Reference		Reference	
Want more	1.50**	[1.37,1.64]	1.52**	[1.39,1.67]
**Region**				
West	Reference		Reference	
Central	1.06	[0.98,1.14]	1.06	[0.98,1.14]
East	1.11*	[1.02,1.20]	1.09*	[1.01,1.19]

Exponentiated coefficients; 95% confidence intervals in brackets
* $p < 0.05$, ** $p < 0.01$

Table S4. Percentage of CMWRAs who engaged in WMC using BDHS 2017-2018

Explanatory variables	% of CMWRAs had WMC	Number	Chi-square value
**Total**	75.8	10,284	
**Age of women**			
15-19	77.3	1,126	38.2***
20-24	78.2	1,939	
25-34	79.4	4,159	
35-49	69.6	3,061	
**Husband's age**			
24<=	78.3	603	60.0***
25-34	79.1	3,162	
35-49	77.9	4,909	
50+	64.4	1,611	
**Years since fisrt cohabitation**			
<3	76.4	1,111	46.9***
3 to 9	79.3	2,821	
10 to 19	79	3,720	
20+	68.2	2,632	
**Parity**			
No child	78.8	941	19.4***
2 child	77.5	5,831	
3+ child	72.4	3,512	
**Age gap**			
0-9 years	78.1	7,119	36.5***
10 to 19 years	71.7	2,850	
20+ years	65.6	316	
**Religion**			
Muslim	75.7	9,189	0.4
Non-Muslim	76.5	1,095	
**Women's education level**			
No	72.7	3,529	11.0***
Primary	77.3	1,121	
<Secondary	77.5	3,888	
>=Secondary	77.7	1,746	
**Husband's education level**			
No	74.8	4,363	2.5
Primary	76	1,293	
<Secondary	77.6	2,537	
>=Secondary	75.6	2,091	
**Women's working status**			
No	74.4	5,046	10.8***
Yes	77.1	5,238	
**Asset quintile**			
Poorest	75.3	2,046	7.4***
Poorer	75.8	2,069	
Middle	72.3	1,913	
Richer	76	2,131	
Richest	79.3	2,126	
**Residence**			
Rural	74.8	7,098	15.6***
Urban	78.1	3,186	
**Person per room**			
<=2	75.3	4,311	2.6
2 to 4	76.6	4,983	
5+	73.8	991	
**BMI**			
Normal	75.8	5,775	1.5

**Explanatory variables**	**% of CMWRAs had WMC**	**Number**	**Chi-square value**
Under-weight	76.2	1,156	
Over-weight	76.4	2,604	
Obese	72.9	750	
**Pregnancy status**			
No/Unsure	76.5	9,690	56.3***
1st trimster	86.4	216	
2nd trimster	68.9	254	
3rd trimster	42.3	125	
**Current contraceptive method**			
Not using	63.9	1,794	116.7***
Short-acting	82.2	6,203	
Tradiional	69.9	1,195	
Long-acting	72	1,092	
**Desire for more child**			
No more	74.1	5,853	60.2***
Want more	80.9	3,519	
**Region**			
West	75	3,575	2.6
Central	77	3,823	
East	75.1	2,886	

Note * $p < 0.05$, ** $p < 0.01$, ***$p<0.001$
